# A Multilevel Approach to Stakeholder Engagement in the Formulation of a Clinical Data Research Network

**DOI:** 10.1097/MLR.0000000000000778

**Published:** 2018-09-13

**Authors:** Alaina P. Boyer, Alecia M. Fair, Yvonne A. Joosten, Rowena J. Dolor, Neely A. Williams, Lisa Sherden, Sarah Stallings, Duane T. Smoot, Consuelo H. Wilkins

**Affiliations:** * Meharry-Vanderbilt Alliance; † Vanderbilt University School of Medicine, Nashville, TN; ‡ Duke University Medical Center, Durham, NC; § Community Partners Network; ∥ Meharry Medical College, Nashville, TN

**Keywords:** stakeholder engagement, clinical data research network, patient-centeredness, community engaged research

## Abstract

**Objectives:**

To ensure meaningful engagement of stakeholders (patients, clinicians, and communities) in developing the Mid-South Clinical Data Research Network (MS-CDRN), we implemented a comprehensive, multilevel approach: (1) identify barriers to involving stakeholders in governance, network design, and implementation; (2) engage stakeholders in priority setting and research topic generation; (3) develop strategies to fully integrate stakeholders in CDRN governance and oversight; and (4) solicit guidance on patient-centered tools and strategies for recruiting research participants.

**Methods:**

We engaged stakeholders: (1) as integral research team members; (2) on oversight and advisory committees; (3) as consultants (using Community Engagement Studios); and (4) through interviews and surveys. We recruited stakeholders from community health centers, churches, barbershops, health fairs, a volunteer registry, and a patient portal. We prioritized recruitment from populations often underrepresented in research.

**Results:**

During the first 18 months, we engaged 5670 stakeholders in developing the MS-CDRN. These were research team members and on governance committees (N=10), consultants (N=58), survey respondents (N=5543), and interviewees (N=59). Stakeholders identified important barriers and facilitators to engagement, developed stakeholder-informed policies, provided feedback on priority topics and research questions, and developed an intake process for data requests and interventional studies that included reviewing for appropriate patient-centeredness, patient engagement, and dissemination.

**Discussion:**

Multilevel stakeholder engagement is a novel systematic approach to developing a meaningful patient-centered and patient-engaged research program. This approach allows ongoing input from highly engaged stakeholders while leveraging focused input from larger, more diverse groups to enhance the patient-centeredness of research and increase relevance to broader audiences.

Engaging patients, consumers, and other community stakeholders in research has emerged as an important mechanism to accelerate the processes used to translate research into practice pragmatically. A 2013 Institute of Medicine report emphasized that stakeholders should be engaged in all phases of clinical and translational research, thus resulting in an increased number of funding announcements requiring stakeholder engagement.^1^ Engaging stakeholders is expected to generate research that is more relevant, increasing public trust and confidence in research while enhancing public participation in research—a central challenge facing clinical research enterprises.

Involving patients and consumers in research is complex, and many investigators struggle to identify approaches for engaging nonresearchers in both timely and meaningful ways.^2^–^4^ Substantial progress has been made in engaging stakeholders in individual research studies and research infrastructure programs like the Clinical and Translational Science Awards.^5^ Yet little is known about methods of involving patients and other stakeholders in large patient-centered research programs like the National Patient-Centered Clinical Research Network (PCORnet). The Mid-South Clinical Data Research Network (MS-CDRN),^6^ led by Vanderbilt University Medical Center, is 1 of 11 CDRNs funded by the Patient-Centered Outcomes Research Institute in phase 1 of PCORnet and encompasses 3 large health systems with >20 million patients nationally. The stakeholder engagement methods required to create a patient-centered research program of this magnitude that is also geographically dispersed has not previously been described.

This paper presents our approach to implementing a comprehensive engagement plan for the MS-CDRN and provides a guiding framework for developing a stakeholder-engaged and patient-centered research network. We describe our methods of stakeholder engagement, the varied backgrounds, and experiences of our stakeholders, and the stakeholder specific input they provided to enrich the formulation of the MS-CDRN.

## METHODS

**FIGURE 1:**
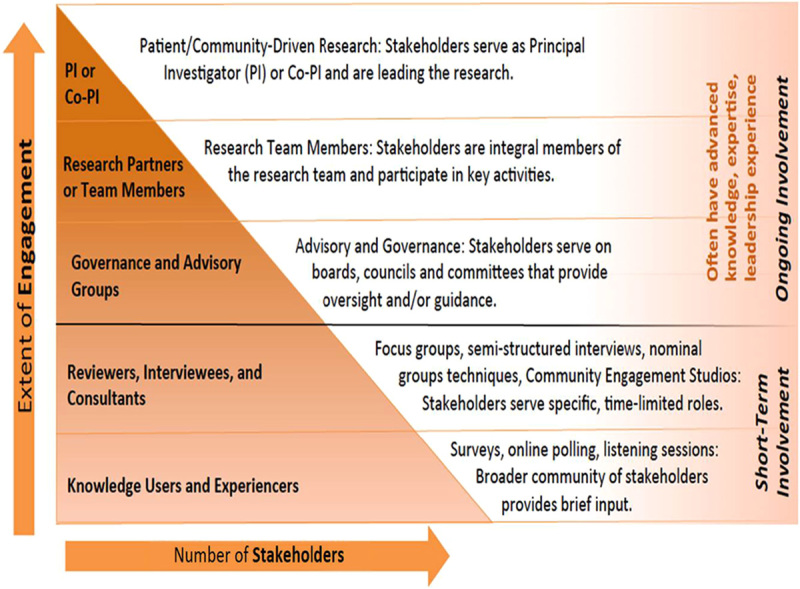
Continuum of Community (Stakeholder) Engagement in Research. A general model by which to build a framework for stakeholder engagement in health research and promote patient-centeredness. Co-PI indicates coprinicipal investigator; PI, prinicipal investigator.

Our approach is guided by a multilevel stakeholder engagement model developed by our team (Fig. 1).^7^ The model incorporates the following key concepts: (1) a number of approaches have successfully involved stakeholders in research; (2) the number of stakeholders engaged and extent of engagement should reflect the goals and aims of the research; (3) stakeholder roles span a continuum from providing brief, targeted input to highly involved, leadership roles and; (4) the training and experience required varies based on stakeholders’ roles. This Continuum of Community Stakeholder Engagement in Research Model was applied to convene the stakeholder members as described below.

Research team: our research team includes a patient investigator (N.A.W.) who participated in all research team activities. The patient investigator provides critical ongoing input to shape the structure and practices of the CDRN by identifying and prioritizing populations, selecting content areas for surveys, suggesting approaches for participant recruitment, providing input on the look and functionality of the website, developing policies and procedures for stakeholder engagement and proposal review, and problem-solving challenges to implementation. The patient investigator was selected based on prior experience as a research advocate and community advisory board member for several research programs.

Oversight Committee: 2 stakeholders representing a local community health center and the state department of public health are members of the Oversight Committee, which is comprised of key leaders who provide informed guidance for overall CDRN performance and offer critical input into problem areas.

Advisory Council: the 8-member Stakeholder Advisory Council is comprised of patients with experience in advisory or leadership roles. Research experience was not a requirement for membership. The patients on the Council represent the health conditions related to the initial CDRN cohorts: sickle cell disease, cardiovascular disease, and obesity.

The Stakeholder Advisory Council met regularly throughout the 18-month start-up period and has provided guidance in the development of policies and operating processes. Orientation was provided to the members to introduce the purpose, goals, and mission of the network. Members received lists of definitions, commonly used acronyms, and rosters of the researchers involved.

Community Engagement Studios (CE Studios)^8^ are a consultative method of engagement to facilitate project-specific input from a panel of health care consumers tailored to the investigator’s population of interest. Panelists share their knowledge and opinions based on their lived experience and provide feedback to the investigator in efforts to improve research processes and implementation. This approach, pioneered by our team, can be used at any stage of research. Six CE Studios were convened with 58 stakeholders to develop the stakeholder engagement plan and provide guidance to the 3 initial cohorts.

Survey respondents and interviewees: our team, including the patient investigator, identified priority populations consisting of racial/ethnic minorities, individuals with multiple chronic conditions, low-income groups, rural and urban residents, and older adults. Our inclusion criteria were broad allowing anyone 18 years or older with capacity to consent to participate in surveys. We used multiple recruitment strategies including in-person engagement at community health centers, minority-owned barbershops, and community health fairs as well as online recruitment using the Vanderbilt patient portal and ResearchMatch,^9^ a volunteer registry.

All surveys and interviews were approved by the Vanderbilt University Medical Center Institutional Review Board.

We surveyed 5063 patients and health care consumers to understand attitudes toward research, barriers to participating in research, and research priorities. Approximately 70% of respondents were women, 23% racial/ethnic minorities and the mean age was 48.1 years (range, 20–94 y; SD, 17.3). Additional demographics are in Table 1.

**TABLE 1:**
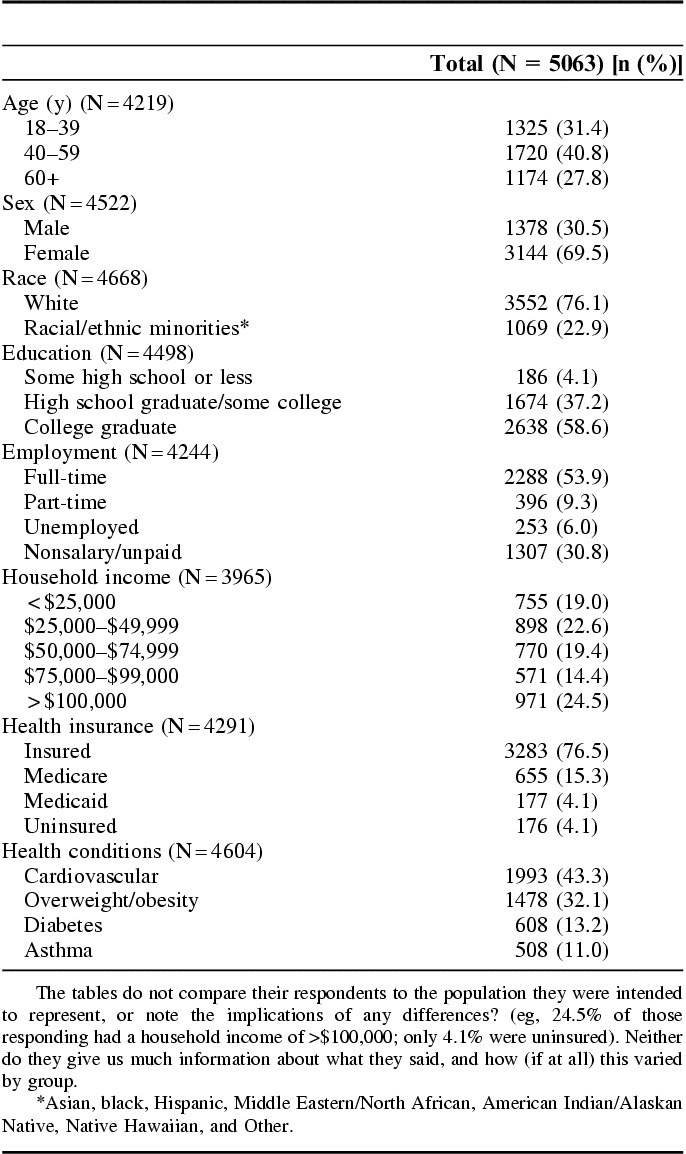
Consumer Survey Demographics

We surveyed 480 health care providers including—primary care and specialty physicians, pharmacists, dentists, physical therapist/occupational therapist/respiratory therapist, psychologists, and licensed social workers) to assess attitudes and interests in participating in a research network and to identify barriers and incentives to participation. In total, 72% of health care providers surveyed were women and 29% were racial/ethnic minorities. Additional demographics are in Table 2. In total, 59 providers were also interviewed to obtain more detailed feedback.

**TABLE 2:**
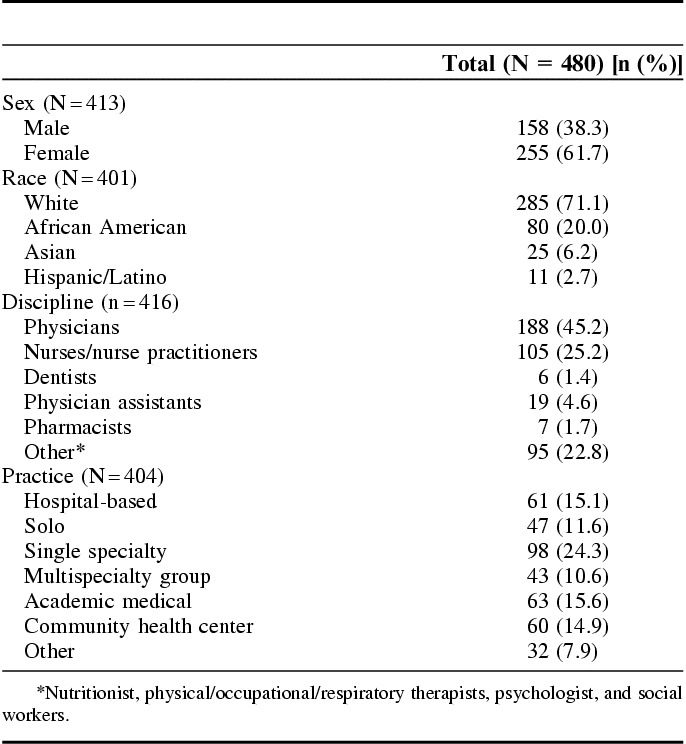
Provider Survey Demographics

To support colearning opportunities for the stakeholder groups described above and enhance communication across levels of engagement (research team, Oversight Committee, Advisory Council, and overall leadership), we created an online shared workspace using Vanderbilt’s Research Organization Collaboration and Knowledge Exchange Toolkit (ROCKET).^10^,^11^ This continues to be an ideal platform for document sharing [leadership meeting presentations, Memoranda of Understanding (MOU), meeting minutes, templates, and planned activities], proposal review, patient-centered resources, glossary of commonly used PCOR/community engaged research and MS-CDRN specific terms and acronyms, and comment boards for feedback and topic generation. This portal does not require Vanderbilt credentials to log in, allowing for easy access to our stakeholders.

## RESULTS

We engaged 5670 patients, clinicians, and community members in developing the MS-CDRN using the multilevel stakeholder engagement model. The vast contributions of these stakeholder groups to enhance our approach to the MS-CDRN infrastructure are reported below.

The stakeholders on the Oversight Committee influenced key decisions for MS-CDRN including phase 2 network expansion, building partnerships with other health care systems, researchers, and organizations, and activity prioritization. A detailed description is available in Table 3.

**TABLE 3:**
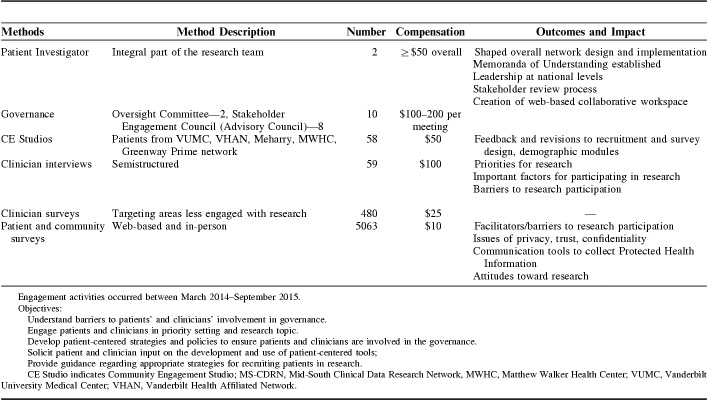
Description of Stakeholder Specific Input and Outcomes on Development of the MS-CDRN

The Stakeholder Advisory Council provided input to the MS-CDRN leadership and investigators to help generate research questions, review research proposals, provide input to researchers on patient engagement and patient-centered outcomes, monitor progress, and help disseminate MS-CDRN information to the broader public. During the early stages, the Stakeholder Advisory Council helped develop policies and processes for integrating stakeholder input into the network, identifying priority populations, and overcoming barriers to engagement. The Advisory Council worked closely with the research team to develop a process to vet all data and research requests made to the MS-CDRN. This process includes a quarterly review of all queries, a review of observational study requests by 2 rotating committee members, and a full committee review of intervention study requests. The intervention study review includes a patient-centeredness plan template for researchers to submit to the committee and patient-centered review criteria.

The group helped develop and subsequently received training on ROCKET, a collaborative communication platform.

Each MS-CDRN cohort research team (healthy weight, coronary heart disease, and Sickle cell) as well as our Stakeholder Engagement team requested stakeholder feedback using the CE Studio process. Six studios were conducted with feedback from 58 stakeholder experts. Feedback to investigators included revisions of recruitment letters to patients, changes to surveys (less technical/medical jargon, more patient-centered questions, more culturally relevant questions), enhanced/tailored recruitment strategies, and changes to protocols (Table 4).

**TABLE 4:**
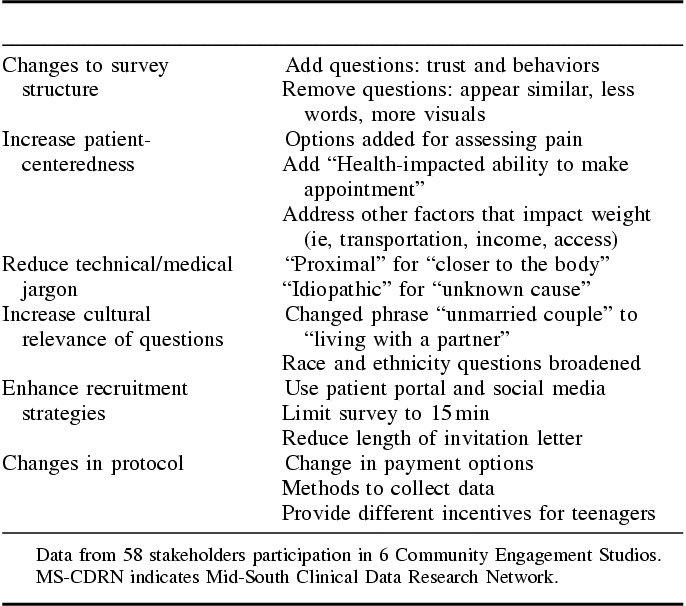
Selected Examples of Changes to Structure of MS-CDRN

The feedback provided by the 5063 patients and health care consumers informed MS-CDRN policies and procedures related to trust, privacy, and confidentiality of patient data, overall interactions with medical research and researchers, and best methods for communication of health information to specific populations. All of the survey items were optional, which was also a suggestion from the stakeholders on our research team and Advisory Council (Table 1).

The 480 health care providers that participated in the survey research identified likely barriers to actively engaging in the CDRN including lack of time, potential negative impact on clinic flow, and additional paperwork. The health care providers’ top priorities for CDRN research were improving management of chronic conditions and preventative health services (Table 2).

## DISCUSSION

We successfully implemented our multilevel stakeholder engagement plan, resulting in substantial input from patients, community members, and health care providers with varied backgrounds and experiences. To our knowledge, stakeholder engagement during the development of a research program has not been previously implemented at this scale. Stakeholders have been engaged in all levels of governance, planning, and implementation of the CDRN including the research team, Stakeholder Advisory Council, and Oversight Committee.

More than 5000 health care consumers gave targeted feedback to determine facilitators and barriers to participation in CDRN research. These surveys captured patients’ concerns surrounding issues of privacy, trust, and confidentiality of clinical data.^6^ This informed MS-CDRN policies and procedures including developing additional measures to protect privacy while fostering transparency in the work of the CDRN. Stakeholder input also helped shape communication between the MS-CDRN and the general public by raising the investigators’ awareness of cultural considerations when addressing or recruiting distinct patient groups and informing the overall interactions between the MS-CDRN and patients/community members. These engagement activities have fostered colearning for researchers and patients and have strengthened communication and linkages among patients, community stakeholders, and the MS-CDRN.

During the 18-month phase I period, we learned a series of effective implementation approaches that will strengthen relationships with the community and health care providers. We identified the following effective approaches: (1) engage stakeholders early in the planning process; (2) provide stakeholders with clear expectations (Memoranda of Understanding were ideal for our approach); (3) provide adequate preparation (orientation, training, resources) for both the stakeholders and the academic team members to ensure meaningful engagement; (4) prioritize effective communication with regular updates and provide explanation of acronyms and research/medical terminology; (5) use established networks such as stakeholder groups affiliated with Clinical Translational Science Awards and Prevention Research Centers; (6) actively engage leaders of patient and health advocacy groups; and (7) leverage providers as trusted agents to facilitate patient engagement.

A comprehensive approach to engagement can be implemented promptly and broadly to elicit the preferences and needs of a broad range of stakeholders. This input can result in important changes to the research infrastructure and process to enhance its relevance and usefulness. Long-term metrics will need to be tracked to determine other impacts such as efficiency of the network and patient-centeredness of the research conducted.
